# Mxi1-0 regulates the growth of human umbilical vein endothelial cells through extracellular signal-regulated kinase 1/2 (ERK1/2) and interleukin-8 (IL-8)-dependent pathways

**DOI:** 10.1371/journal.pone.0178831

**Published:** 2017-06-02

**Authors:** Weiling Wu, Zhenzhen Hu, Feng Wang, Hao Gu, Xiuqin Jiang, Jinjin Xu, Xi Zhan, Datong Zheng, Zhengdong Zhang

**Affiliations:** 1 Children’s Health Center, The Second Hospital, Nanjing Medical University, Nanjing, Jiangsu, P. R. China; 2 Clinical Molecular Diagnostic Laboratory, The Second Hospital, Nanjing Medical University, Nanjing, Jiangsu, P. R. China; 3 The Second Clinical School, Nanjing Medical University, Nanjing, Jiangsu, P. R.China; 4 Center for Vascular and inflammatory Diseases, University of Maryland School of Medicine, Baltimore, MD, United States of America; 5 State Key Laboratory of Reproductive Medicine, Nanjing Medical University, Nanjing, Jiangsu, P. R.China; Medical College of Wisconsin, UNITED STATES

## Abstract

Mxi1 plays an important role in the regulation of cell proliferation. Mxi1-0, a Mxi1 isoform, has a different N-terminal amino acid sequence, intracellular location and expression profile from Mxi1. However, the precise role of Mxi1-0 in cell proliferation and the molecular mechanism underlying its function remain poorly understood. Here, we showed that Mxi1-0 suppression decreased the proliferation of human umbilical vein endothelial cells (HUVECs) along with cell accumulation in the G2/M phase. Mxi1-0 suppression also significantly decreased the expression and secretion of interleukin (IL-8). Neutralizing IL-8 in conditioned medium (CM) from Mxi1-0-overexpressed HUVECs significantly eliminated CM-induced proliferation of HUVECs. In addition, Mxi1-0 suppression significantly decreased the activity of MAP kinase ERK1/2. Treatment of HUVECs with U0126, an ERK1/2 signaling inhibitor, attenuated autocrine production of IL-8 induced by Mxi1-0 overexpression. On the other hand, Mxi1-0 overexpression-induced IL-8 increased the level of phosphorylated ERK1/2 in HUVECs, and such increasing was diminished in cells incubated with CM, which neutralized with anti-IL-8 antibody. Taken together, our results suggest that Mxi1-0 regulates the growth of HUVECs via the IL-8 and ERK1/2 pathways, which apparently reciprocally activate each other.

## Introduction

Mxi1-0 is a transcription factor containing a basic helix-loop-helix leucine zipper (bHLHzip) and belongs to the Myc-Max-Mad transcriptional network [1, 2]. Mxi1-0 protein is the translational product of a transcript derived from an alternative exon of the Mxi1 gene and hence has a N-terminal sequence of 66 amino acids that is different from Mxil [3]. In contrast to Mxi1, Mxi1-0 is unable to inhibit c-Myc-dependent transcriptional events [4, 5], resides in distinct cellular compartments and has an expression profile different from Mxi1 [3]. A recent study has shown that overexpression of Mxi1-0 promotes proliferation [6]. However, the molecular mechanism for Mxi1-0-indcued cell proliferation remains undefined.

Interleukin-8 (IL-8) is a proinflammatory CXC chemokine associated with the promotion of neutrophil chemotaxis and degranulation [7, 8]. Previous studies have suggested that secretion of IL-8 from cancer cells can aggravate the proliferation and survival of cancer cells, in part by autocrine signaling pathways [9, 10]. IL-8 has also been recognized as an angiogenic factor. Secretion of IL-8 from cancer cells can activate endothelial cells to promote angiogenesis [7, 11]. In addition, IL-8 is secreted by endothelial cells, thereby enhancing endothelial cell survival, proliferation, and angiogenesis [7, 12]. Although the role for IL-8 in mediating endothelial cell survival, proliferation and angiogenesis has been strongly suggested, the upstream signaling events associated with IL-8 expression and secretion have not been well characterized.

Mitogen-activated protein kinases (MAPKs), including extracellular signal-regulated kinase (ERK), c-Jun N-terminal kinase (JNK) and p38 MAPK, have been implicated in the induction of IL-8 expression and secretion [13–15]. It has been shown that Mxi1 inactivates MAPK signaling in different cell types [16, 17] and that IL-8 is up-regulated in the cells in the absence of Mxi1 [17, 18]. However, the effects of Mxi1-0 on the activation of MAPKs and IL-8 expression in endothelial cells have not been studied. This study was aimed to investigate whether Mxi1-0 and IL-8 might be involved in the proliferation of human umbilical vein endothelial cells (HUVECs), and to define signaling events associated with IL-8 expression and secretion in HUVECs. Our results indicate that Mxi1-0 regulates the growth of HUVECs via the activation of IL-8 and ERK1/2 pathways.

## Materials and methods

### Cell culture and collection of conditional media

Human umbilical vein endothelial cells (HUVECs) were obtained from Keygen biotech (Nanjing, China). Cells were maintained in DMEM:F12K medium (Gibco, GrandIsland, NY) containing 10% (v/v) fetal bovine serum (FBS) (Hyclone, Logan, UT), 100 units penicillin/mL, 100 mg/mL streptomycin,0.1mg/mL heparin and 0.05mg/mL endothelial cell growth supplement (ECGS) in a humidified atmosphere at 37°C with 5% CO_2_. Confluent cultures between passages 2–3 were used in this study to minimize age-dependent variation in the level of apoptosis. Conditioned medium (CM) was collected in sterile conditions followed by centrifugation at 3000 rpm for 20 min at 4°C, and then stored at −80°C for further use. CM1 and CM2 are self conditioned media collected from the culture medium of HUVECs-transfected with empty vector and Mxi1-0 plasmid, respectively. Complete medium alone without cells was incubated under the same experimental conditions served as control.

### Small interfering RNA (siRNA)

For gene knockdown, siRNA duplexes specific for Mxi1-0 (On-Target Plus: 5’- CACCAGCGAGAACUCGAUGGATT-3’, 5’-CAGCGAGAACUCGAUGGAGAATT-3’, and 5’-CACUUUUCUGCAGAACGUGCATT-3’), and IL-8 (On-Target Plus: 5’-GGUGCAGUUUUGCCAAGGATT-3’, 5’-GCCAGAUGCAAUACAAGAUTT-3’ and 5’-GAAGAGGGCUGAGAAUUCATT- 3’) were transfected into HUVECs using Lipofectamine 2000 reagent following the manufacturer's instructions. The knockdown efficiency was evaluated 48 h after transfection by measuring mRNA and protein expression in the lysates of transfected cells with qRT-PCR and Western blot, respectively.

### Enzyme linked-immuno sorbent assay (ELISA)

IL-8 concentrations in the cell supernatant were quantified by using IL-8 enzyme-linked immunosorbent assay (ELISA) kit (Shanghai Joyee Biotechnics Co., Ltd, Shanghai, China) according to the manufacturer’s instructions.

### qRT-PCR analyses

Total RNA was extracted, and cDNA was synthesized, as described previously [19]. Quantitative real-time PCR was performed using the SYBR Premix Ex Taq II (Takara Biotechnology Co., Dalian, China). 2−ΔCT method was used to calculate gene expression levels. The level of each RNA was normalized to that of the housekeeping gene GAPDH. The sequences of the oligonucleotides (Invitrogen, Shanghai, China) were as follows: IL-8, (forward, 5’-CTGGCCGTGGCTCTCTTG-3’ and reverse, 5’-CCTTGGCAAAACTGCACCTT-3’); GAPDH (forward, 5’-GCACCGTCAAGGCTGAGAAC-3’ and reverse, 5’-TGGTGAAGACGCCAGTGGA-3’).

### Western blot analysis

Cellular lysates and western blotting assays were performed as previously depicted [20–22]. The following antibodies were used: mouse anti-β-actin (Sigma, St. Louis, MO, USA), rabbit anti-Mxi1-0 (Invitrogen, Carlsbad, CA), rabbit anti-ERK1/2, anti-phospho-ERK1/2 (Thr202/Tyr204), anti-phospho-p38, anti-p38, anti-phospho-JNK, anti-JNK, anti- phospho-Cdk1(Tyr15) and anti-phospho-Chk1(Ser345) (Cell Signaling Technology, Boston, MA, USA), rabbit anti-CyclinB1(Proteintech,Wuhan, China). Antiserum against Mxi1 and Mxi1-0 were raised in rabbits by injection of Keyhole Limpet Hemocyanin (KLH)–conjugated, synthetic peptides (CEAAEFLERRE for Mxi1, GKRGRPRKEARCE for Mxi1-0), corresponding to amino acids 13–23 of Mxi1 and amino acids 2–14 of Mxi1-0, respectively. Antibodies were affinity-purified by Invitrogen (Carlsbad, CA, USA).

### Cell viability assay

Cell viability was measured by CCK-8 assay. Briefly, cells transfected with different siRNAs were seeded onto 96-well plates in triplicate wells (3×10^3^cells/well) in culture medium without antibiotics. Cell viability was determined at the indicated times by the CCK-8 kit according to the manufacturer’s protocol. Optical density (OD) was read at 450 nm using the MD SpectraMax Plus384 microplate reader (ELx800, BioTek Instruments, Inc., Vermont, USA). Three independent experiments were done.

### Cell proliferation assay

Cell proliferation was assessed by EdU incorporation. Proliferating cells were determined by the kFluor488-EdU kit (Keygen biotech) according to the manufacturer’s protocol and were identified by green staining. For quantification analysis, the number of proliferating cells in five randomly chosen fields was counted.

### Cell cycle assay

Cells were synchronized at the G0/G1 phase by culturing in serum-free medium for 24h, then transfected with Mxi1-0 siRNA or negative control siRNA for 48h. Cells were then stimulated with 10%FBS for 24h and detached by trypsin digestion, fixed with ice-cold 70% ethanol for 4 h, and stained with 50 μg/ml propidium iodide (PI) in the presence of RNase A at37°C for 30 min. Intracellular DNA content was analyzed using a FACSCalibur flow cytometer (BD Biosciences, San Jose, CA, USA).

### Cell apoptosis assay

Cell apoptosis was evaluated by flow cytometry using an Annexin-V-FITC Apoptosis Detection Kit (BD Bioscience, USA) according to the manufacturer’s instructions. Briefly, the cells were harvested and washed twice in phosphate-buffered saline (PBS) and resuspended in 500 μl of binding buffer. A volume of 5 μl of Annexin-V-FITC and 5 μl of PI was added and mixed gently, and the cells were stained in the dark for 15 min at room temperature. The cells were analyzed immediately by flow cytometry and analyzed using Modfit V3.0.

### Immunofluorescence

Cells on coverslips were fixed in 4% formaldehyde for 20 min, permeabilized in 0.2% Triton X-100 for 10 min, and incubated with a primary antibody against IL-8 overnight at 4°C. The cells were then incubated with Alexa Fluor-568–conjugated secondary antibody for 1 h in the dark, after which, the nuclei were stained with DAPI (4, 6-diamidino-2-phenylindole) for 10 min. Images were acquired using a fluorescence microscope (Leica DM2500, Wetzlar, Germany).

### Statistical analysis

Statistical analysis was carried out using the SPSS statistical software package version 17 (SPSS Inc., Chicago, IL). Student's t test was used to analyze the differences between two groups. When comparisons between multiple groups were carried out, one-way ANOVA followed by SNK tests were employed. Statistical significance was considered at P<0.05.

## Results

### Suppression of Mxi1-0 expression in human umbilical vein endothelial cells by siRNA

**Fig 1:**
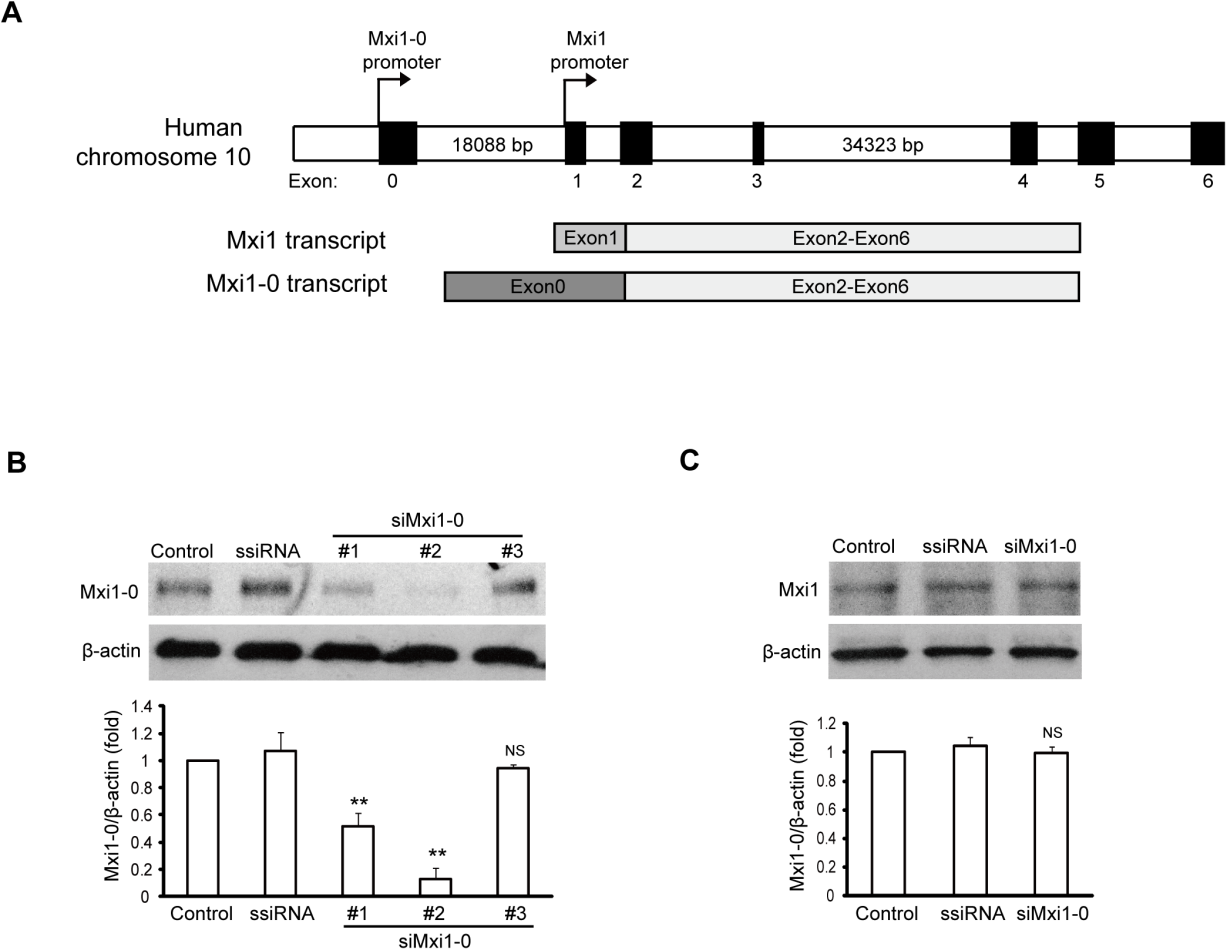
Inhibition of Mxi1-0 expression in HUVECs by siRNAs. (A) Schematic presentations for the genomic organization of the human Mxi1 locus (upper), and isoforms Mxi1-0 and Mxi1 (lower). (B) HUVECs were transfected with the appropriate siRNAs as described in Materials and Methods. After transfection, the level of Mxi1-0 protein was analyzed by Western blot analysis. (C) Mxi1 expression in HUVECs after transfection with Mxi1-0 siRNA (siMxi1-0) or scrambled siRNA (ssiRNA) was measured by Western blot analysis. **P<0.01 vs. scrambled siRNA. NS: no statistically difference vs. scrambled siRNA.

Mxi1-0 is a splicing isoform of Mxi1 by using an alternative first exon (exon 0, Fig 1A). Therefore, three types of siRNAs specifically against Mxi1-0 were designed by having a sequence corresponding to exon 0. Western blot was used to evaluate the activity of Mxi1-0 siRNAs with polyclonal antibodies raised against synthetic peptides corresponding to translational products of exon 0 and exon 1, respectively. While type 1 and type 2 siRNAs were able to suppress Mxi1-0 protein expression by 48.33% and 86.67%, respectively, type 3 siRNA showed no detectable effect on Mxi1-0 protein expression (Fig 1B). Thus, type 2 siRNA (siMxi1-0) was chosen to knockdown Mxi1-0 for the rest study. Also, this siRNA did not inhibit Mxi1 protein expression (Fig 1C), indicative of its specificity.

### Mxi1-0 is required for the proliferation of HUVECs

**Fig 2:**
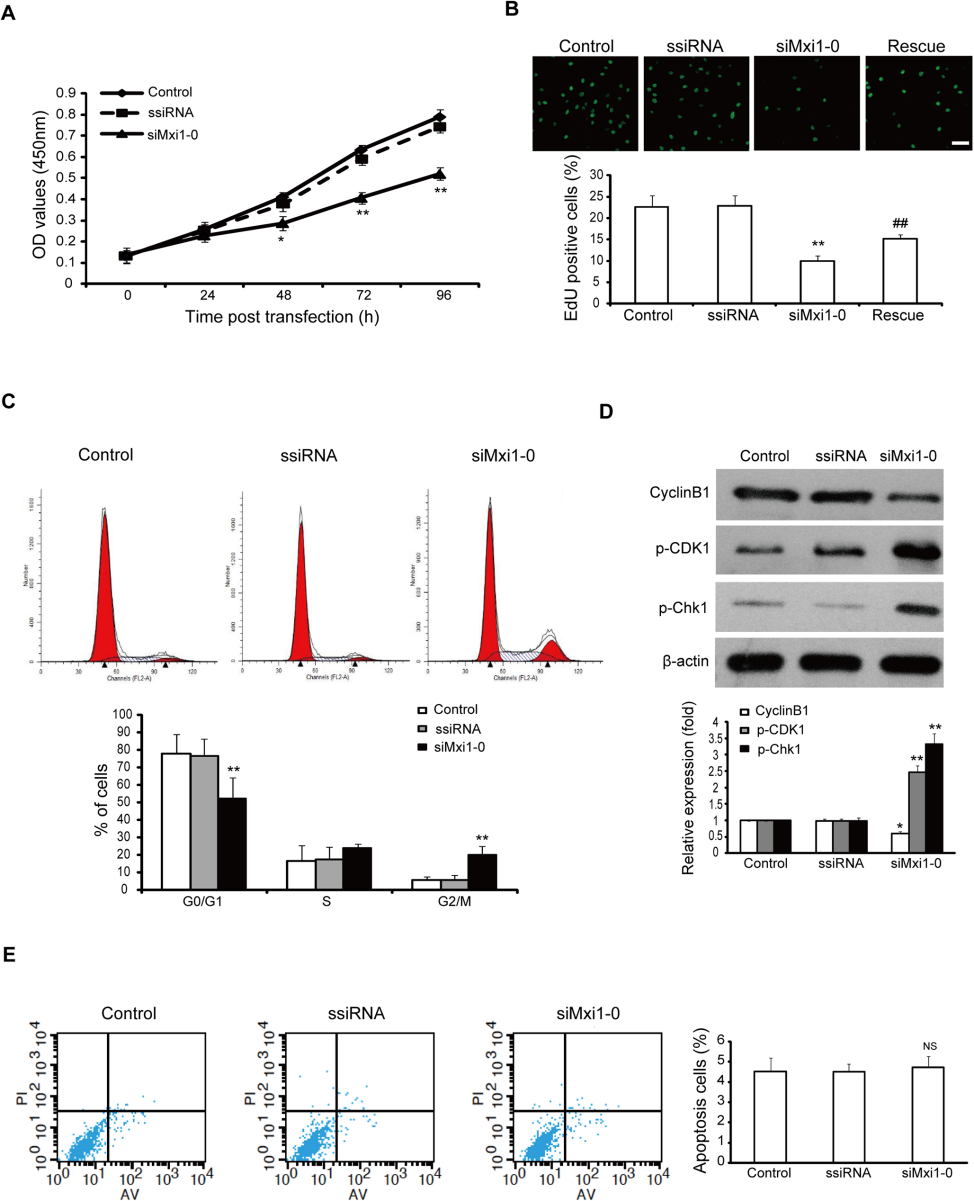
Knockdown of Mxi1-0 inhibits the growth of HUVECs *in vitro*. (A) HUVECs were treated with siMxi1-0 or ssiRNA, and the growth of the cells at the indicated times was estimated by using CCK-8 assay. *P<0.05, **P<0.01 vs. scrambled siRNA. (B) Upper: EdU staining of HUVECs transfected with siMxi1-0 or rescued with Mxi1-0 plasmid, Lower: the statistical analyses of upper. Scale bar represents 200μm. **P<0.01 vs. scrambled siRNA, ## P<0.01 vs. Mxi1-0 siRNA. (C) The transfected cells were harvested and stained with propidium iodide (PI), and then quantified for the cycle distribution by flow cytometry. **P<0.01 vs. scrambled siRNA. (D) Western blot analysis of CyclinB1, p-CDK1, and p-Chk1 protein expressions in the siRNA-transfected cells. *P<0.05, **P<0.01 vs. scrambled siRNA. (E) Flow cytometry analysis of annexin V/PI staining was performed to evaluate the percentage of apoptotic cells. NS: no statistically difference vs. scrambled siRNA.

We first examined the effect of Mxi1-0 suppression on the growth of HUVECs by CCK-8 assay. As shown in Fig 2A, transfection with Mxi1-0 siRNA reduced proliferation of HUVECs when assessed daily during a 4-day period. Mxi1-0 knockdown cells exhibited diminished proliferative capacity, which was rescued by re-expressing Mxi1-0 (Fig 2B).

To determine whether the growth-inhibitory effect of Mxi1-0 suppression could result from changes in the cell cycle, we measured cellular DNA content by flow cytometry. The results showed that Mxi1-0 suppression resulted in the accumulation of the proportion of cells in the G2/M phase from 5.87% to 20.04% (Fig 2C). To elucidate the molecular mechanism underlying G2/M phase arrest, we examined the expression levels of G2/M cell cycle regulatory proteins. There was a significant decrease in the level of cyclinB1 in the Mxi1-0 siRNA-transfected cells, however, the protein expression of p-CDK1 (Tyr15) and p-Chk1 (Ser345) increased (Fig 2D).

To determine whether the growth-inhibitory effect of Mxi1-0 suppression could also result from cell death, we measured cell apoptosis by flow cytometry by double labeling with Annexin V and PI. The results showed that Mxi1-0 suppression did not cause any significant change in the apoptosis of HUVECs (Fig 2E). Taken together, these findings suggest that Mxi1-0 suppression inhibited proliferation and cell cycle progression in HUVECs.

### Mxi1-0 regulates the proliferation of HUVECs through production of interleukin-8 (IL-8)

**Fig 3:**
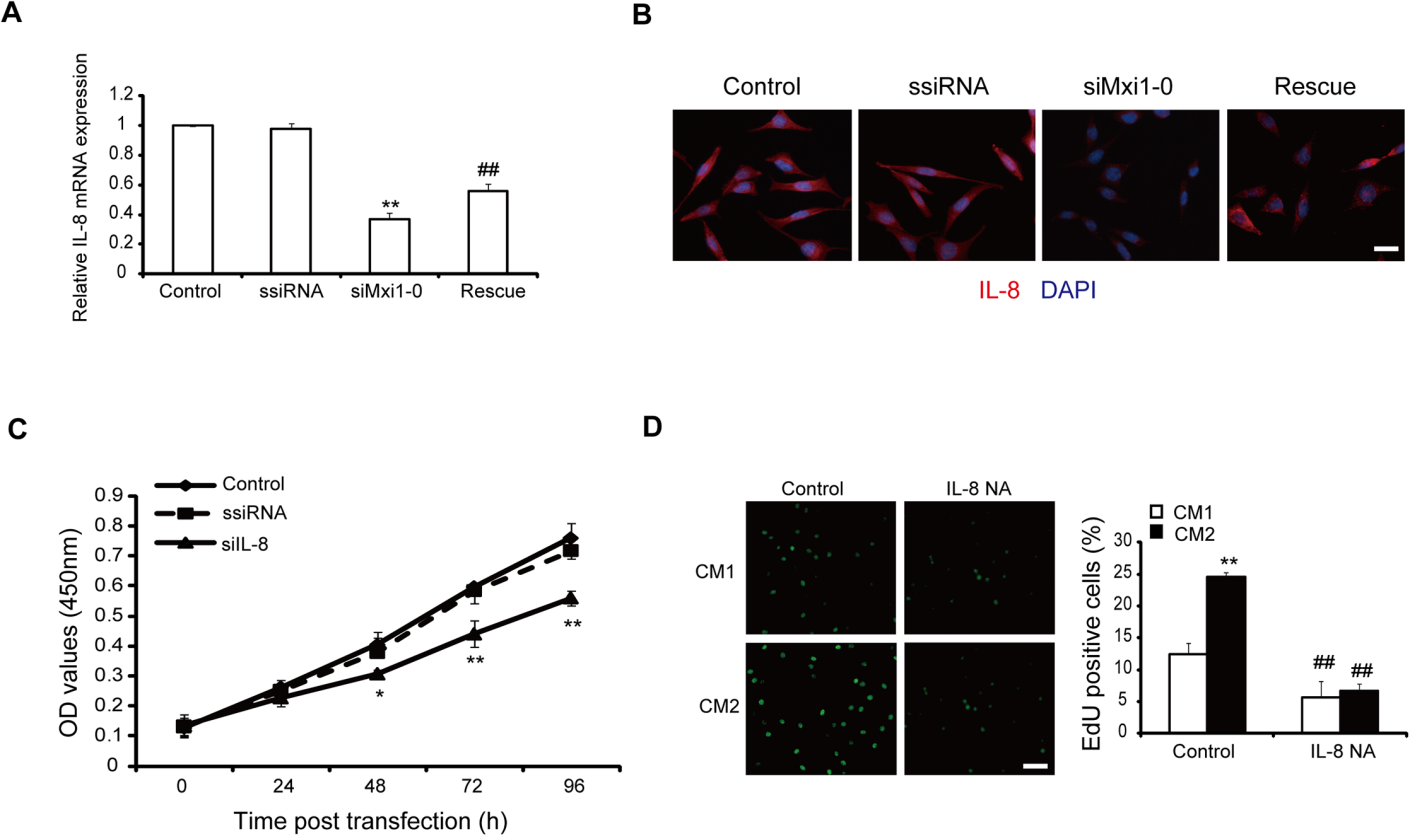
IL-8 is required for Mxi1-0-mediated proliferation of HUVECs. (A& B) Effect of the Mxi1-0 siRNA (siMxi1-0) on the expression of IL-8.HUVECs were transfected with siMxi1-0 or rescued with Mxi1-0 plasmid were subjected to qRT-PCR (A) and Immunofluorescence (B). Scale bar represents 100μm. *P<0.05, **P<0.01 vs. scrambled siRNA. (C) HUVECs were transfected with IL-8 siRNA (siIL-8) or scrambled siRNA (ssiRNA), and the growth of the cells at the indicated times was estimated by using CCK-8 assay. *P<0.05, **P<0.01 vs. scrambled siRNA. (D) HUVECs were incubated with CM1, CM2 or CM1/CM2 pre-treated with neutralising antibody against IL-8 (IL-8 NA). The proliferation of HUVECs was estimated by using EdU assay. Left: EdU staining of HUVECs incubated with CM, Right: the statistical analyses of left. Scale bar represents 200μm. **P<0.01 vs.CM1, ^##^ P<0.01 vs.Control.

Previous studies showed that IL-8 is up-regulated in the absence of Mxi1, the isoform of Mxi1-0 [17, 18]. Thus, we examined the effect of Mxi1-0 suppression on IL-8 expression in HUVECs. Mxi1-0 suppression reduced IL-8 markedly in the mRNA and protein levels, which was rescued by re-expressing Mxi1-0 (Fig 3A and 3B). The results from CCK-8 assay showed that IL-8 suppression significantly inhibited proliferation of HUVECs (Fig 3C). As IL-8 can aggravate the proliferation of endothelial cells in an autocrine manner [23], we thought that the growth-inhibitory effect of Mxi1-0 suppression might be caused by decreasing in the autocrine production of IL-8. To test this hypothesis, we first treated HUVECs with the conditioned medium (CM) collected from the cells transfected with empty vector (CM1) or with Mxi1-0 plasmid (CM2). The results showed that the number of EdU staining positive cells was ~2.1 fold higher in CM2 than in CM1 (Fig 3D), suggesting the proliferation potentiating effect of CM2 on HUVECs. Then, we treated CM1 and CM2 with neutralising antibody against IL-8 followed by EdU assay. Our results showed inhibition of proliferation in HUVECs when cultured in CM1 and CM2 treated with neutralising antibody against IL-8 (Fig 3D). Moreover, the magnitude of this inhibition was higher in CM2 treated with neutralising antibody against IL-8 (Fig 3D), suggesting IL-8 as a major mediator of Mxi1-0 overexpression-induced proliferation in HUVECs. Taken together, these results suggest that Mxi1-0 regulates the proliferation of HUVECs through autocrine production of IL-8.

### Mxi1-0 regulates autocrine production of IL-8 through the ERK1/2 signaling pathway

**Fig 4:**
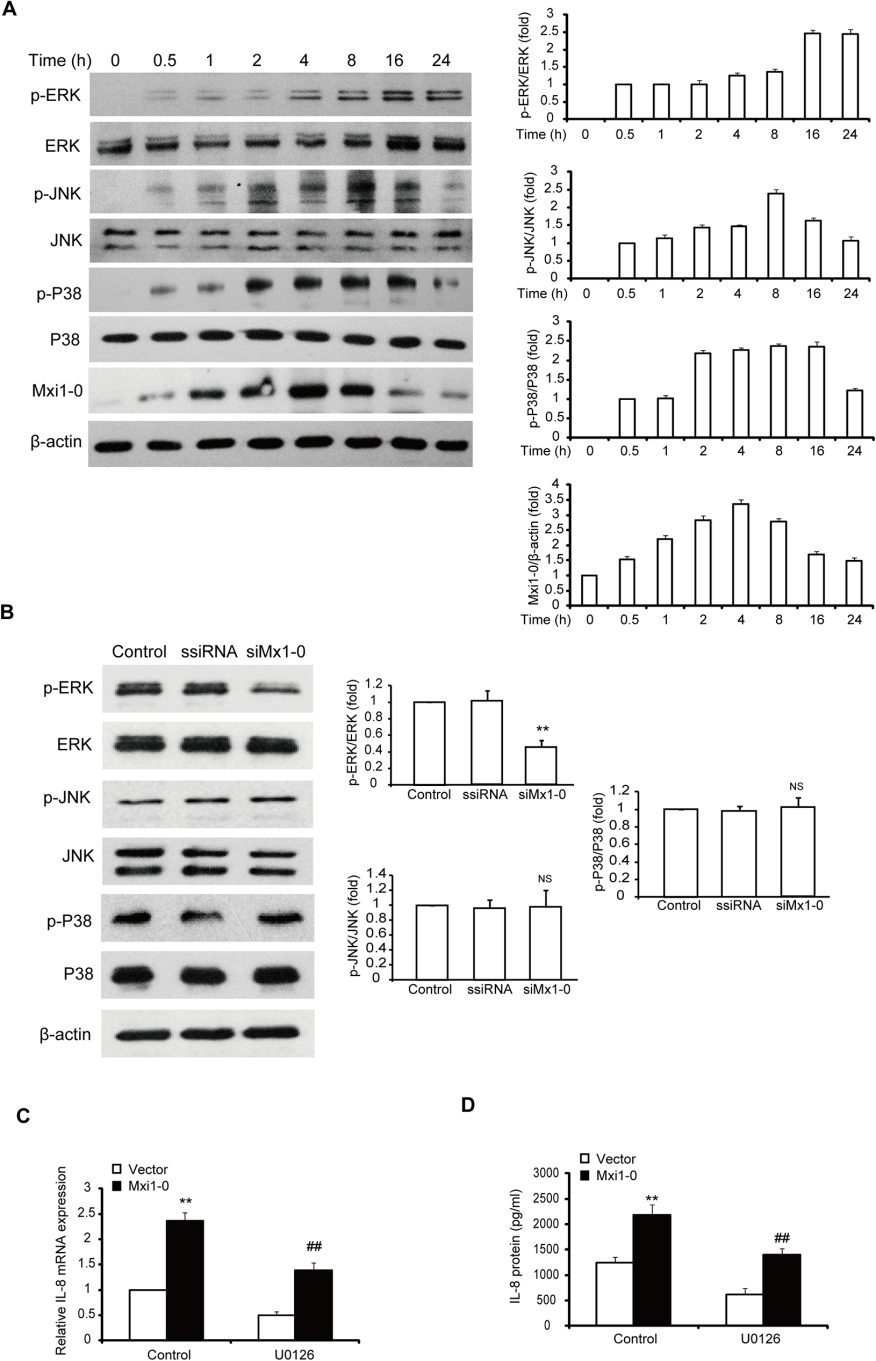
Mxi1-0 regulates autocrine production of IL-8 in HUVECs via EKR1/2-dependent pathway. (A) Mxi1-0 expression and MAPK activation during HUVECs proliferation. HUVECs were cultured in the absence of serum for 24 h before stimulation with 20% serum for the indicated amount of time. The lysates were fractionated and analyzed by Western blotting with the indicated antibodies. (B) Effects of Mxi1-0 knockdown on the activation of MAPK pathway. The levels of phosphorylated ERK1/2, p38 and JNK in ssiRNA or siMxi1-0-transfected cells were measured by Western blot. **P<0.01 vs. scrambled siRNA. NS: no statistically difference vs. scrambled siRNA. (C& D) The effect of MEK inhibitor U0126 on Mxi1-0 overexpression-induced IL-8 in HUVECs. Mxi1-0-overexpressed cells were pre-treated with 10 μM U0126 for 2 h. The expression of IL-8 mRNA was estimated by qRT-PCR (C), and the level of secreted IL-8 was measured by ELISA (D), respectively. **P<0.01 vs.Vector, ##P<0.01 vs. Control.

As MAPKs are implicated in IL-8 expression and secretion [24–26], we explored the possible role of MAPKs in the Mxi1-0-mediated autocrine production of IL-8. First, we examined the expression of Mxi1-0 and the levels of phosphorylated ERK1/2, p38 and JNK MAPKs during proliferation in HUVECs. The results showed that when compared to non-proliferation cells (0 h), Mxi1-0 expression significantly increased and reached at its peak at 4 h after serum stimulation and then reduced (Fig 4A). The phosphorylation of ERK1/2 also significantly increased after serum stimulation with maximal activation at 16 h (Fig 4A). The phosphorylation of JNK reached at its peak at 8 h after serum stimulation (Fig 4A). Alternatively, its phosphorylation was markedly decreased at 24 h (Fig 4A). P38 maintains high activation at 2 to 16 h after serum stimulation and its phosphorylation was also markedly decreased at 24 h (Fig 4A). Then, we examined the effect of Mxi1-0 suppression on the activation of ERK1/2, p38 and JNK MAPKs. As shown in Fig 4B, Mxi1-0 suppression resulted in marked decrease in the phosphorylation of ERK1/2, but no changes in the phosphorylation of p38 and JNK. To examine whether Mxi1-0-induced autocrine production of IL-8 is ERK1/2 activation dependent, we treated Mxi1-0-overexpressed HUVECs with U0126, an ERK1/2 signaling inhibitor. The U0126-treated cells displayed less IL-8 expression and secretion than did control cells as determined by qRT-PCR (Fig 4C) or ELISA (Fig 4D). Therefore, Mxi1-0 regulates autocrine of IL-8 in HUVECs via EKR1/2-dependent pathway.

### Reciprocal activation between IL-8 and ERK1/2 pathway in HUVECs

**Fig 5:**
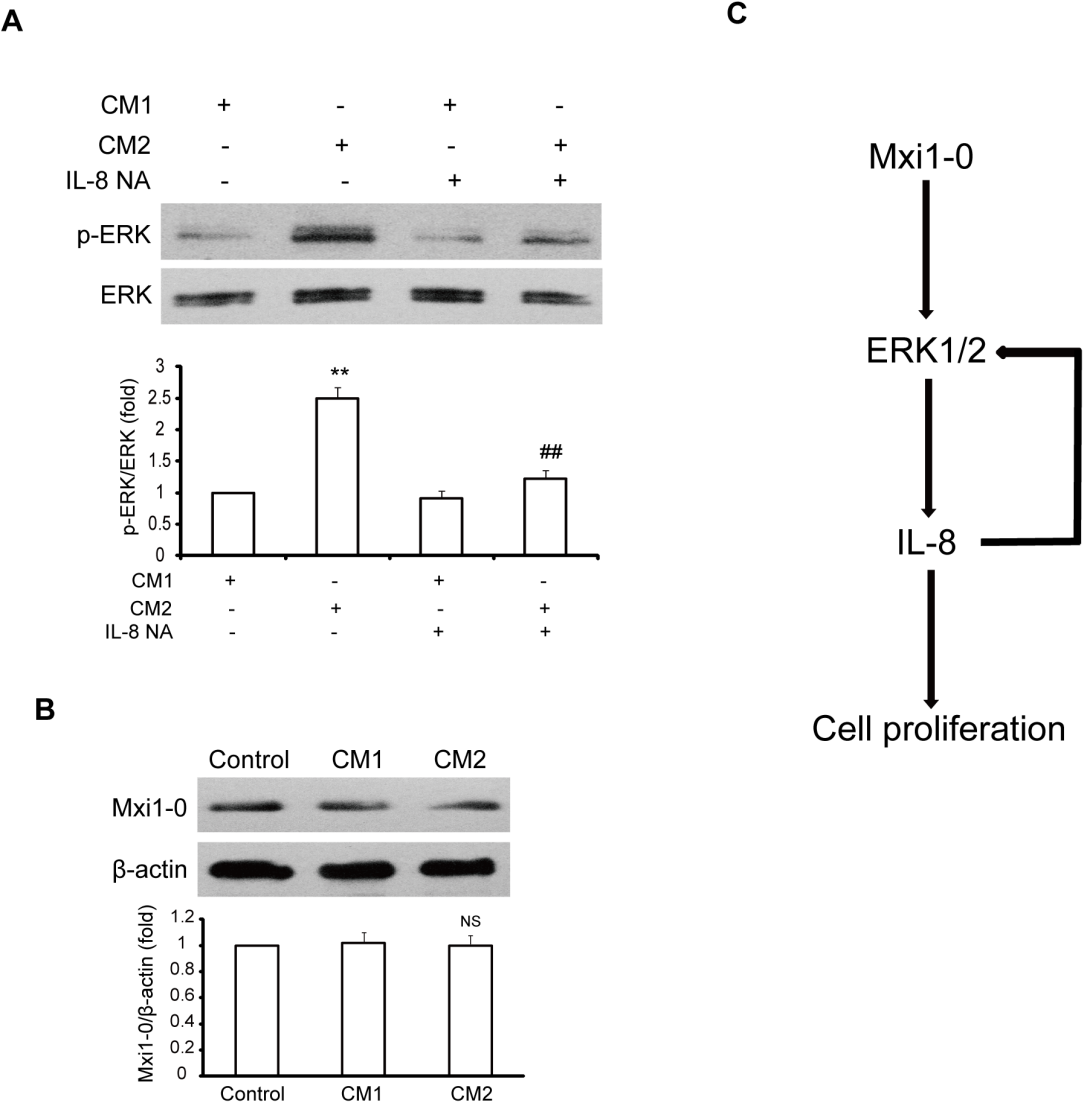
IL-8 regulates the activation of ERK1/2 in HUVECs. (A) HUVECs were incubated with CM1, CM2, or CM1/CM2 pre-treated with neutralising antibody against IL-8 (IL-8 NA). Phosphorylation of ERK1/2 was detected by Western blot. **P<0.01 vs.CM1, ^##^ P<0.01 vs.CM2 without IL-8 NA. (B) Mxi1-0 expression in HUVECs incubated with CM1 or CM2 was measured by Western blot. NS: no statistically difference vs. CM1. (C) Schematic diagram shows the mechanism underlying Mxi1-0-mediated proliferation of HUVECs: Mxi1-0 activates ERK1/2 signaling pathway that upregulates autocrine production of IL-8, in turn, the autocrine IL-8 perpetuates further ERK1/2 activation, which forms a positive feedback loop and promotes Mxi1-0-mediated proliferation of HUVECs.

It has been previously reported that the IL-8 triggers the activation of the ERK1/2 signaling pathway [27]. To examine whether the ERK1/2 signaling pathway in HUVECs was also modulated by autocrinally produced IL-8 in our system, we incubated HUVECs with the conditioned medium collected from HUVECs that transfected with empty vector (CM1) or with Mxi1-0 plasmid (CM2), and then treated CM1 and CM2 with neutralising antibody against IL-8 followed by ERK1/2 phosphorylation analysis. The results showed that the phosphorylation of ERK1/2 in the cells incubated with CM2 was greatly increased compared to the cells treated with CM1 (Fig 5A). The increased phosphorylation of ERK1/2 was significantly inhibited in the cells incubated with CM2 treated with neutralising antibody against IL-8 (Fig 5A). On the other hand, there is not any significant change in the expression of Mxi1-0 in the cells incubated with CM2 (Fig 5B). Therefore, the data herein indicate that IL-8 and ERK1/2 pathway reciprocally each other in Mxi1-0-mediated proliferation of HUVECs.

## Discussion

Mxi1-0 is a novel Mxi1 isoform that fails to negatively regulate c-Myc function and its role in proliferation is not well understood. In the present study, the proliferative capacity of HUVECs was significantly diminished when Mxi1-0 expression was inhibited. Our findings are consistent with a previous study, which shows that induction of Mxi1-0 leads to increased proliferation in neuroblastoma cells [6]. Therefore, the effect of Mxi1-0 on cell proliferation is contrary to Mxi1, which generally inhibits cell growth [16, 28, 29]. Based on this, the mechanism underlying the effect of Mxi1-0 on proliferation was investigated.

Two checkpoints, G1/S transition and G2/M transition, play crucial roles in maintaining cell proliferation [30]. In our study, we found that Mxi1-0 suppression induced significant G2/M phase cell cycle arrest. Proportion of G2/M phase cell nearly 4 fold in Mxi1-0 siRNA-transfected cells compared with negative control siRNA-transfected cells. Cell cycle is tightly controlled by cyclin/cyclin-dependent kinase (Cdks) complexes. Cyclin B/CDK1 complex drives the G2/M transition and controls processes during mitosis [31, 32]. Entering the M phase correctly needs activation of CDK1 which involves combination of CDK1 and cyclin B1 [32]. The initial activation of cyclin B1/CDK1 involves CDK1 dephosphorylation at Thr14 and Tyr15 by Cdc25C [32, 33]. Decreased phosphatase activity of Cdc25C leads to inactivation of cyclin B1/CDK1 [33, 34]. Chk1, a protein kinase of the CAMLK family, negatively regulate cell cycle progression during unperturbed cell cycles [35, 36]. Activated Chk1 can phosphorylate and inactivate Cdc25C via phosphorylation and 14-3-3 binding [37]. Our further investigation revealed that these cell cycle regulatory proteins were involved in Mxi1-0 suppression induced cell cycle arrest. In the present study, the expression of cyclin B1 was decreased in Mxi1-0 siRNA-transfected cells. Mxi1-0 suppression also caused accumulation of Tyr15-phosphorylated CDK1, which represents inactivation of CDK1. Moreover, expression of phosphorylated Chk1 was also increased after Mxi1-0 suppression. According to the results mentioned above, Mxi1-0 suppression induced G2/M phase arrest in HUVECs by the downregulation of cyclin B1 and inactivation of CDK1 and Chk1.

Further study on the mechanism of Mxi1-0 suppression induced growth-inhibition showed that IL-8 signaling was involved. We focused our investigation on IL-8 because previous studies showed that Mxi-1 is involved in IL-8 induction [17, 18]. In the present study, IL-8 expression was inhibited by Mxi1-0 suppression. When IL-8 expression was blocked, the proliferation of HUVECs was dramatically diminished, indicating that Mxi1-0 suppression induced growth-inhibition may due to a decrease of IL-8. It has been reported that autocrine IL-8 can aggravate the proliferation and survival of endothelial cells [19]. In our system, cell proliferation rate of HUVECs was accelerated after CM2 treatment compared with CM1. At the same time, the effect of CM2 was inhibited by neutralising antibody against IL-8. Therefore, autocrine IL-8 directly activates the proliferation of HUVECs transfected with Mxi1-0. Based on this, we conclude that autocrinally produced IL-8 is an important modulator for the Mxi1-0-mediated proliferation of HUVECs. A previous study showed that the expression level of IL-8 in human microvascular endothelial cells (HMEC-1) is related to Mxi1/c-Myc interaction [18]. Although it has high homology with Mxi1, Mxi1-0 has not been shown to inhibit c-Myc-dependent transcriptional events [4, 5]. Furthermore, Mxi1-0 resides in distinct cellular compartments and has an expression profile different from Mxi1 [3]. Therefore, we postulate that Mxi1-0 regulates proliferation in a c-Myc-independent manner in HUVECs.

In the investigation for the mechanisms involved in the Mxi1-0-mediated production of IL-8, we found that Mxi1-0 suppression reduced markedly phosphorylation of ERK1/2, and that pharmacological inhibition of ERK1/2 significantly inhibited autocrine production of IL-8 in Mxi-0-overexpressed HUVECs. Thus, Mxi1-0-mediated regulation of IL-8 synthesis in HUVECs is dependent on the activation of ERK1/2. This finding is consistent with previous reports that MAPKs are implicated in IL-8 expression and secretion [24–26] and that there is a correlation between Mxi1-0 and MAPKs [16–18]. Our previous study demonstrated that Mxi1-0 could regulate ROS generation [19], which is required for ERK1/2 activation [38, 39]. The role of ROS that function downstream of Mxi1-0 and in controlling ERK1/2 activation in our system remains to be determined. Our results also showed that Mxi1-0 suppression had any effect on the activation of p38 MAPK, which has been involved in the inhibition of IL-8 secretion in HEK293T cells by Mxi1 [17], and induction of IL-8 expression in HUVECs mediated by Urotensin II [24]. These differences may reflect the different functions between Mxi1-0 and Mxi1 or different cellular settings for these studies. As the change in the activation of ERK1/2 is consistently observed, we conclude that ERK1/2 plays an important role in the induction of IL-8 in Mxi1-0-overexpressed HUVECs. Although the mechanism for ERK1/2 to regulate IL-8 expression is unknown, ERK1/2 acts as a downstream target of the IL-8 signaling cascade [27]. Consistent with this notion, we found that the CM derived from Mxi1-0-overexpressed HUVECs treated with neutralising antibody against IL-8 showed reduced ability to induce ERK1/2 phosphorylation, while it has no effect on the expression of Mxi1-0. This suggests that Mxi1-0 overexpression-induced IL-8 perpetuated further ERK1/2 activation in HUVECs.

In summary, our results indicate that Mxi1-0 activates ERK1/2 signaling pathway that upregulates autocrine production of IL-8, in turn, the autocrine IL-8 perpetuates further ERK1/2 activation, which forms a positive feedback loop and promotes Mxi1-0-mediated proliferation of HUVECs (Fig 5C). These findings are of potential pathophysiological importance for understanding the integration of angiogenesis-related signaling.
